# Ethyl 2-(4-hy­droxy-3-meth­oxy­phen­yl)-1-[3-(2-oxopyrrolidin-1-yl)prop­yl]-1*H*-benzimidazole-5-carboxyl­ate monohydrate

**DOI:** 10.1107/S1600536811052391

**Published:** 2011-12-10

**Authors:** Yeong Keng Yoon, Mohamed Ashraf Ali, Tan Soo Choon, Safra Izuani Jama Asik, Ibrahim Abdul Razak

**Affiliations:** a Institute for Research in Molecular Medicine, Universiti Sains Malaysia, Minden 11800, Penang, Malaysia; b School of Physics, Universiti Sains Malaysia, 11800 USM, Penang, Malaysia

## Abstract

In the title compound, C_24_H_27_N_3_O_5_·H_2_O, the essentially planar benzimidazole ring system [maximum deviation = 0.020 (1) Å] forms dihedral angles of 54.10 (11) and 67.79 (6)°, respectively, with the mean plane of pyrrolidin-2-one ring and the benzene ring. The pyrrolidin-2-one ring adopts an envelope conformation with one of the methylene C atoms at the flap. An intra­molecular C—H⋯π inter­action is observed. In the crystal, O—H⋯O and O—H⋯N hydrogen bonds link the two components into a double-tape structure along the *a* axis. The crystal packing is further stabilized by weak π–π stacking [centroid–centroid distance = 3.6632 (9) Å] and C—H⋯O inter­actions.

## Related literature

For the biological activity of benzimidazole derivatives, see: Rao *et al.* (2002 ▶); Thakurdesai *et al.* (2007 ▶); Dubey & Sanyal (2010 ▶). For related structures, see: Yoon *et al.* (2011 ▶). For the ring conformation, see: Cremer & Pople (1975) ▶.
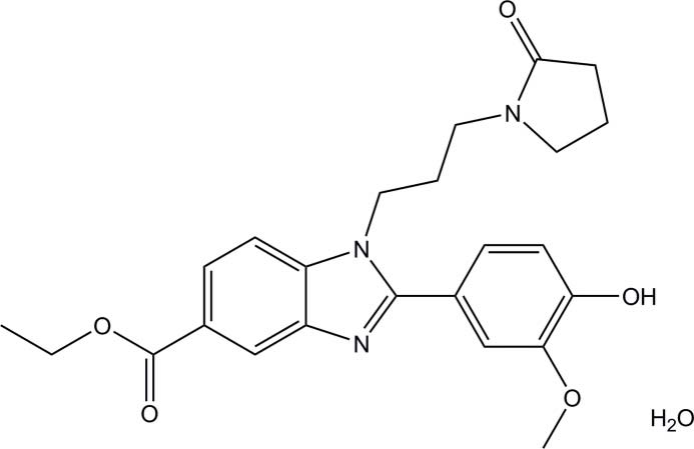


## Experimental

### 

#### Crystal data

C_24_H_27_N_3_O_5_·H_2_O
*M* *_r_* = 455.50
Triclinic, 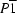

*a* = 9.7460 (8) Å
*b* = 10.0436 (8) Å
*c* = 12.6072 (10) Å
α = 85.737 (1)°
β = 89.684 (2)°
γ = 70.238 (1)°
*V* = 1157.91 (16) Å^3^
*Z* = 2
Mo *K*α radiation
μ = 0.10 mm^−1^
*T* = 296 K
0.43 × 0.32 × 0.16 mm

#### Data collection

Bruker APEXII DUO CCD area-detector diffractometer
Absorption correction: multi-scan (*SADABS*; Bruker, 2009 ▶) *T*_min_ = 0.960, *T*_max_ = 0.985
18195 measured reflections
6686 independent reflections
4485 reflections with *I* > 2σ(*I*)
*R*_int_ = 0.029

#### Refinement

*R*[*F* ^2^ > 2σ(*F* ^2^)] = 0.055
*wR*(*F* ^2^) = 0.185
*S* = 1.07
6686 reflections
312 parameters
H atoms treated by a mixture of independent and constrained refinement
Δρ_max_ = 0.30 e Å^−3^
Δρ_min_ = −0.26 e Å^−3^

### 

Data collection: *APEX2* (Bruker, 2009 ▶); cell refinement: *SAINT* (Bruker, 2009 ▶); data reduction: *SAINT*; program(s) used to solve structure: *SHELXTL* (Sheldrick, 2008 ▶); program(s) used to refine structure: *SHELXTL*; molecular graphics: *SHELXTL*; software used to prepare material for publication: *SHELXTL* and *PLATON* (Spek, 2009 ▶).

## Supplementary Material

Crystal structure: contains datablock(s) global, I. DOI: 10.1107/S1600536811052391/is5022sup1.cif

Structure factors: contains datablock(s) I. DOI: 10.1107/S1600536811052391/is5022Isup2.hkl

Supplementary material file. DOI: 10.1107/S1600536811052391/is5022Isup3.cml

Additional supplementary materials:  crystallographic information; 3D view; checkCIF report

## Figures and Tables

**Table 1:** Hydrogen-bond geometry (Å, °) *Cg*4 is the centroid of the C8–C13 benzene ring.

*D*—H⋯*A*	*D*—H	H⋯*A*	*D*⋯*A*	*D*—H⋯*A*
O1*W*—H2*W*1⋯O2^i^	0.85 (3)	2.06 (3)	2.879 (2)	164 (2)
O1*W*—H1*W*1⋯N1^ii^	0.95 (3)	1.90 (3)	2.8416 (19)	176 (2)
O4—H1*O*4⋯O1*W*	0.88 (3)	1.80 (3)	2.675 (2)	169 (3)
C16—H16*A*⋯O5^iii^	0.96	2.44	3.380 (3)	166
C20—H20*B*⋯*Cg*4	0.97	2.86	3.750 (3)	153

Symmetry codes: (i) 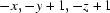; (ii) 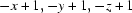; (iii) 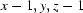.

**Fig. 1.**
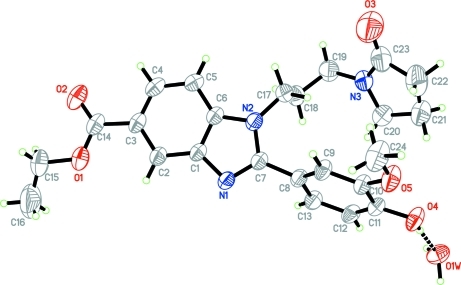
The structure of the title compound, showing 50% probability displacement ellipsoids. Hydrogen atoms are shown as spheres of arbitrary radius.

**Fig. 2.**
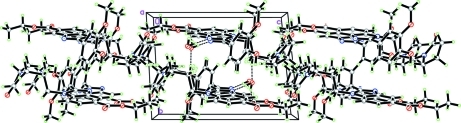
The crystal packing, viewed along the a-axis. Hydrogen bonds are shown as dashed lines.

## supplementary crystallographic information

### Comment

Benzimidazole derivatives are of wide interest because of their diverse biological activities and various clinical applications. This ring system is present in numerous anti-HIV (Rao *et al.*, 2002), anti-inflammatory (Thakurdesai *et al.*, 2007) and anthelmintics (Dubey and Sanyal, 2010) drugs. As part of our ongoing structural studies of benzimidazole derivatives (Yoon *et al.*, 2011), we now report the structure of the title compound.

In the title compound, Fig. 1, the benzimidazole (N1/N2/C1–C7) ring system is essentially planar with a maximum deviation of 0.020 (1) Å for atom N2. Dihedral angles of benzimidazole, (N1/N2/C1–C7) ring with the mean plane of pyrrolidin-2-one ring (N3/C20–C23) and the 2-methoxyphenol (C8–C13) ring are 54.10 (11) and 67.79 (6)°, respectively. The pyrrolidin-2-one ring adopts an envelope conformation with puckering parameters of Q = 0.148 (3) Å and φ = 78.5 (10)° (Cremer & Pople, 1975) with atom C21 at the flap, deviating by 0.095 (3) Å. In the crystal packing (Fig. 2), intermolecular O1W—H2W1···O2, O4—H1O4···O1W and O1W—H1W1···N1 hydrogen bonds link the molecules into a double-tape structure running along the *a* axis. There is a π–π stacking interaction between the benzene (C1–C6; centroid *Cg*3) rings with a *Cg*3···*Cg*3^iv^ distance of 3.6632 (9) Å [symmetry code: (iv) -*x*, -*y*, -*z* + 1]. The crystal packing is further stabilized by a C16—H16A···O5 hydrogen bond and a weak C20—H20B···*Cg*4 interaction (Table 1), where *Cg*4 is the centroid of the benzene (C8–C13) ring.

### Experimental

Ethyl 3-amino-4-(3(2-oxopyrrolidin-1yl)propylamino)benzoate (0.84 mmol) and sodium metabisulfite adduct of 4-hydroxy-3-metoxybenzaldehyde (1.68 mmol) were dissolved in DMF. The reaction mixture was reflux at 130 °C for 2 hrs. After completion, the reaction mixture was diluted in ethyl acetate (20 mL) and washed with water (20 mL). The organic layer was collected, dried over Na_2_SO_4_ and the evaporated in vacuo to yield the product. The product was recrystallized from ethyl acetate.

### Refinement

The O-bound H atom was located in a difference Fourier map and refined freely [O—H = 0.88 (3), 0.85 (3) and 0.95 (3) Å]. The remaining H atoms were positioned geometrically and refined using a riding model, with C—H = 0.93–0.97 Å. The *U*_iso_(H) values were constrained to be 1.5*U*_eq_ (methyl-H atom) and 1.2*U*_eq_ (other H atoms). The rotating model group was applied for the methyl group.

### Crystal data

**Table:**

C_24_H_27_N_3_O_5_·H_2_O	*Z* = 2
*M**_r_* = 455.50	*F*(000) = 484
Triclinic, *P*1	*D*_x_ = 1.306 Mg m^−^^3^
Hall symbol: -P 1	Mo *K*α radiation, λ = 0.71073 Å
*a* = 9.7460 (8) Å	Cell parameters from 4470 reflections
*b* = 10.0436 (8) Å	θ = 2.5–29.5°
*c* = 12.6072 (10) Å	µ = 0.10 mm^−^^1^
α = 85.737 (1)°	*T* = 296 K
β = 89.684 (2)°	Block, brown
γ = 70.238 (1)°	0.43 × 0.32 × 0.16 mm
*V* = 1157.91 (16) Å^3^	

### Data collection

**Table:**

Bruker APEXII DUO CCD area-detector diffractometer	6686 independent reflections
Radiation source: fine-focus sealed tube	4485 reflections with *I* > 2σ(*I*)
graphite	*R*_int_ = 0.029
φ and ω scans	θ_max_ = 30.1°, θ_min_ = 1.6°
Absorption correction: multi-scan (*SADABS*; Bruker, 2009)	*h* = −13→13
*T*_min_ = 0.960, *T*_max_ = 0.985	*k* = −14→14
18195 measured reflections	*l* = −17→17

### Refinement

**Table:**

Refinement on *F*^2^	Primary atom site location: structure-invariant direct methods
Least-squares matrix: full	Secondary atom site location: difference Fourier map
*R*[*F*^2^ > 2σ(*F*^2^)] = 0.055	Hydrogen site location: inferred from neighbouring sites
*wR*(*F*^2^) = 0.185	H atoms treated by a mixture of independent and constrained refinement
*S* = 1.07	*w* = 1/[σ^2^(*F*_o_^2^) + (0.0974*P*)^2^ + 0.1068*P*] where *P* = (*F*_o_^2^ + 2*F*_c_^2^)/3
6686 reflections	(Δ/σ)_max_ < 0.001
312 parameters	Δρ_max_ = 0.30 e Å^−^^3^
0 restraints	Δρ_min_ = −0.26 e Å^−^^3^

### Special details

**Table:**

Geometry. All esds (except the esd in the dihedral angle between two l.s. planes) are estimated using the full covariance matrix. The cell esds are taken into account individually in the estimation of esds in distances, angles and torsion angles; correlations between esds in cell parameters are only used when they are defined by crystal symmetry. An approximate (isotropic) treatment of cell esds is used for estimating esds involving l.s. planes.
Refinement. Refinement of F^2^ against ALL reflections. The weighted R-factor wR and goodness of fit S are based on F^2^, conventional R-factors R are based on F, with F set to zero for negative F^2^. The threshold expression of F^2^ > 2sigma(F^2^) is used only for calculating R-factors(gt) etc. and is not relevant to the choice of reflections for refinement. R-factors based on F^2^ are statistically about twice as large as those based on F, and R- factors based on ALL data will be even larger.

### Fractional atomic coordinates and isotropic or equivalent isotropic displacement parameters (Å^2^)

**Table:**

	*x*	*y*	*z*	*U*_iso_*/*U*_eq_	
O1	−0.17090 (17)	0.13373 (19)	0.18216 (12)	0.0762 (4)	
O2	−0.35072 (14)	0.11827 (17)	0.28604 (13)	0.0702 (4)	
O3	0.1298 (3)	0.2186 (2)	1.00505 (19)	0.1183 (8)	
O4	0.64165 (13)	0.40228 (14)	0.73582 (10)	0.0538 (3)	
O5	0.64019 (13)	0.14280 (12)	0.76404 (10)	0.0552 (3)	
N1	0.17611 (13)	0.25473 (14)	0.43596 (10)	0.0416 (3)	
N2	0.09463 (13)	0.23337 (13)	0.60105 (10)	0.0401 (3)	
N3	0.12756 (16)	0.41105 (17)	0.89776 (11)	0.0529 (4)	
C1	0.04959 (15)	0.21973 (15)	0.43129 (12)	0.0379 (3)	
C2	−0.02368 (16)	0.19669 (16)	0.34357 (13)	0.0412 (3)	
H2A	0.0100	0.2035	0.2749	0.049*	
C3	−0.14954 (15)	0.16306 (15)	0.36302 (13)	0.0421 (3)	
C4	−0.20115 (16)	0.15385 (17)	0.46613 (14)	0.0456 (4)	
H4A	−0.2863	0.1325	0.4759	0.055*	
C5	−0.12959 (16)	0.17542 (17)	0.55325 (13)	0.0443 (3)	
H5A	−0.1636	0.1686	0.6218	0.053*	
C6	−0.00288 (15)	0.20810 (15)	0.53349 (12)	0.0378 (3)	
C7	0.19826 (15)	0.26220 (15)	0.53799 (12)	0.0386 (3)	
C8	0.31855 (15)	0.29914 (16)	0.58469 (12)	0.0391 (3)	
C9	0.42362 (16)	0.19835 (15)	0.65042 (12)	0.0419 (3)	
H9A	0.4206	0.1068	0.6616	0.050*	
C10	0.53227 (15)	0.23334 (15)	0.69912 (12)	0.0397 (3)	
C11	0.53539 (16)	0.37285 (16)	0.68324 (12)	0.0399 (3)	
C12	0.43197 (17)	0.47134 (16)	0.61670 (13)	0.0436 (3)	
H12A	0.4346	0.5630	0.6051	0.052*	
C13	0.32404 (17)	0.43507 (16)	0.56685 (13)	0.0437 (3)	
H13A	0.2557	0.5019	0.5216	0.052*	
C14	−0.23434 (18)	0.13642 (17)	0.27471 (15)	0.0492 (4)	
C15	−0.2499 (4)	0.1148 (4)	0.0897 (2)	0.1125 (11)	
H15A	−0.2417	0.0159	0.0893	0.135*	
H15B	−0.3524	0.1707	0.0942	0.135*	
C16	−0.1953 (5)	0.1561 (5)	−0.0036 (3)	0.1330 (14)	
H16A	−0.2453	0.1380	−0.0633	0.199*	
H16B	−0.0930	0.1034	−0.0068	0.199*	
H16C	−0.2095	0.2556	−0.0055	0.199*	
C17	0.07375 (17)	0.25520 (17)	0.71444 (12)	0.0440 (3)	
H17A	0.1679	0.2234	0.7510	0.053*	
H17B	0.0174	0.1990	0.7447	0.053*	
C18	−0.00473 (18)	0.41025 (18)	0.73095 (13)	0.0489 (4)	
H18A	−0.1024	0.4388	0.7003	0.059*	
H18B	0.0462	0.4669	0.6936	0.059*	
C19	−0.01470 (19)	0.4404 (2)	0.84774 (15)	0.0574 (4)	
H19A	−0.0725	0.5393	0.8537	0.069*	
H19B	−0.0644	0.3828	0.8853	0.069*	
C20	0.2172 (3)	0.4956 (3)	0.86944 (19)	0.0786 (7)	
H20A	0.1655	0.5951	0.8783	0.094*	
H20B	0.2485	0.4851	0.7964	0.094*	
C21	0.3479 (3)	0.4349 (4)	0.9482 (2)	0.0957 (9)	
H21A	0.4390	0.4191	0.9113	0.115*	
H21B	0.3420	0.4996	1.0028	0.115*	
C22	0.3385 (3)	0.2990 (3)	0.9957 (2)	0.0910 (8)	
H22A	0.3541	0.2909	1.0721	0.109*	
H22B	0.4112	0.2194	0.9659	0.109*	
C23	0.1884 (3)	0.3012 (2)	0.96930 (17)	0.0675 (5)	
C24	0.6452 (2)	−0.0001 (2)	0.7783 (2)	0.0696 (6)	
H24A	0.7301	−0.0550	0.8204	0.104*	
H24B	0.6497	−0.0381	0.7102	0.104*	
H24C	0.5593	−0.0041	0.8141	0.104*	
O1W	0.63716 (17)	0.67068 (15)	0.71300 (12)	0.0565 (3)	
H2W1	0.556 (3)	0.736 (3)	0.7004 (18)	0.073 (7)*	
H1W1	0.703 (3)	0.695 (2)	0.6663 (19)	0.076 (7)*	
H1O4	0.630 (3)	0.493 (3)	0.7238 (18)	0.075 (7)*	

### Atomic displacement parameters (Å^2^)

**Table:**

	*U*^11^	*U*^22^	*U*^33^	*U*^12^	*U*^13^	*U*^23^
O1	0.0729 (9)	0.1176 (13)	0.0612 (9)	−0.0609 (9)	−0.0102 (7)	−0.0141 (8)
O2	0.0485 (7)	0.0835 (10)	0.0904 (10)	−0.0369 (7)	−0.0121 (7)	−0.0103 (8)
O3	0.1466 (19)	0.0967 (14)	0.1279 (17)	−0.0717 (14)	−0.0518 (14)	0.0407 (12)
O4	0.0477 (6)	0.0476 (7)	0.0739 (8)	−0.0271 (5)	−0.0160 (6)	−0.0001 (6)
O5	0.0519 (6)	0.0420 (6)	0.0730 (8)	−0.0197 (5)	−0.0242 (6)	0.0066 (5)
N1	0.0363 (6)	0.0502 (7)	0.0435 (7)	−0.0221 (5)	−0.0036 (5)	−0.0006 (5)
N2	0.0382 (6)	0.0431 (7)	0.0430 (7)	−0.0192 (5)	−0.0022 (5)	−0.0026 (5)
N3	0.0548 (8)	0.0617 (9)	0.0458 (8)	−0.0234 (7)	−0.0013 (6)	−0.0086 (6)
C1	0.0316 (6)	0.0379 (7)	0.0462 (8)	−0.0151 (6)	−0.0041 (5)	0.0001 (6)
C2	0.0367 (7)	0.0432 (8)	0.0460 (8)	−0.0171 (6)	−0.0052 (6)	−0.0011 (6)
C3	0.0338 (7)	0.0362 (7)	0.0577 (9)	−0.0139 (6)	−0.0086 (6)	−0.0017 (6)
C4	0.0337 (7)	0.0423 (8)	0.0641 (10)	−0.0177 (6)	−0.0005 (7)	−0.0005 (7)
C5	0.0383 (7)	0.0458 (8)	0.0514 (9)	−0.0186 (6)	0.0033 (6)	−0.0004 (6)
C6	0.0337 (6)	0.0348 (7)	0.0457 (8)	−0.0131 (5)	−0.0038 (5)	0.0002 (5)
C7	0.0346 (7)	0.0388 (7)	0.0443 (8)	−0.0155 (6)	−0.0037 (6)	−0.0003 (6)
C8	0.0364 (7)	0.0419 (7)	0.0423 (8)	−0.0180 (6)	−0.0025 (6)	−0.0020 (6)
C9	0.0419 (7)	0.0351 (7)	0.0528 (9)	−0.0189 (6)	−0.0077 (6)	−0.0008 (6)
C10	0.0365 (7)	0.0366 (7)	0.0473 (8)	−0.0145 (6)	−0.0073 (6)	0.0006 (6)
C11	0.0360 (7)	0.0406 (7)	0.0478 (8)	−0.0191 (6)	0.0000 (6)	−0.0047 (6)
C12	0.0432 (8)	0.0378 (7)	0.0545 (9)	−0.0208 (6)	−0.0018 (6)	0.0015 (6)
C13	0.0417 (7)	0.0418 (8)	0.0491 (8)	−0.0176 (6)	−0.0066 (6)	0.0039 (6)
C14	0.0426 (8)	0.0444 (8)	0.0645 (11)	−0.0203 (7)	−0.0120 (7)	−0.0002 (7)
C15	0.124 (2)	0.192 (3)	0.0685 (16)	−0.112 (3)	−0.0155 (15)	−0.0232 (18)
C16	0.184 (4)	0.162 (3)	0.088 (2)	−0.108 (3)	−0.050 (2)	0.003 (2)
C17	0.0454 (8)	0.0480 (8)	0.0404 (8)	−0.0189 (7)	−0.0021 (6)	−0.0004 (6)
C18	0.0408 (8)	0.0543 (9)	0.0485 (9)	−0.0118 (7)	−0.0045 (6)	−0.0056 (7)
C19	0.0480 (9)	0.0665 (11)	0.0564 (10)	−0.0157 (8)	0.0059 (8)	−0.0154 (8)
C20	0.0812 (14)	0.1101 (19)	0.0640 (12)	−0.0598 (14)	−0.0060 (11)	0.0033 (12)
C21	0.0717 (15)	0.157 (3)	0.0758 (15)	−0.0603 (18)	−0.0095 (12)	−0.0113 (16)
C22	0.0764 (15)	0.103 (2)	0.0856 (17)	−0.0190 (15)	−0.0253 (13)	−0.0107 (14)
C23	0.0776 (13)	0.0626 (12)	0.0628 (12)	−0.0239 (11)	−0.0135 (10)	−0.0052 (9)
C24	0.0683 (12)	0.0404 (9)	0.0982 (16)	−0.0193 (9)	−0.0323 (11)	0.0140 (9)
O1W	0.0552 (7)	0.0477 (7)	0.0723 (9)	−0.0254 (6)	0.0088 (6)	−0.0028 (6)

### Geometric parameters (Å, °)

**Table:**

O1—C14	1.315 (2)	C11—C12	1.382 (2)
O1—C15	1.460 (2)	C12—C13	1.391 (2)
O2—C14	1.214 (2)	C12—H12A	0.9300
O3—C23	1.216 (3)	C13—H13A	0.9300
O4—C11	1.3575 (17)	C15—C16	1.382 (5)
O4—H1O4	0.88 (3)	C15—H15A	0.9700
O5—C10	1.3621 (18)	C15—H15B	0.9700
O5—C24	1.418 (2)	C16—H16A	0.9600
N1—C7	1.317 (2)	C16—H16B	0.9600
N1—C1	1.3944 (17)	C16—H16C	0.9600
N2—C7	1.3750 (19)	C17—C18	1.515 (2)
N2—C6	1.3774 (18)	C17—H17A	0.9700
N2—C17	1.465 (2)	C17—H17B	0.9700
N3—C23	1.341 (3)	C18—C19	1.521 (2)
N3—C20	1.437 (3)	C18—H18A	0.9700
N3—C19	1.453 (2)	C18—H18B	0.9700
C1—C2	1.395 (2)	C19—H19A	0.9700
C1—C6	1.395 (2)	C19—H19B	0.9700
C2—C3	1.394 (2)	C20—C21	1.542 (3)
C2—H2A	0.9300	C20—H20A	0.9700
C3—C4	1.399 (2)	C20—H20B	0.9700
C3—C14	1.483 (2)	C21—C22	1.481 (4)
C4—C5	1.373 (2)	C21—H21A	0.9700
C4—H4A	0.9300	C21—H21B	0.9700
C5—C6	1.398 (2)	C22—C23	1.494 (3)
C5—H5A	0.9300	C22—H22A	0.9700
C7—C8	1.4808 (19)	C22—H22B	0.9700
C8—C13	1.386 (2)	C24—H24A	0.9600
C8—C9	1.394 (2)	C24—H24B	0.9600
C9—C10	1.3830 (19)	C24—H24C	0.9600
C9—H9A	0.9300	O1W—H2W1	0.85 (3)
C10—C11	1.411 (2)	O1W—H1W1	0.95 (3)
			
C14—O1—C15	117.16 (17)	C16—C15—H15B	109.4
C11—O4—H1O4	109.2 (16)	O1—C15—H15B	109.4
C10—O5—C24	117.13 (13)	H15A—C15—H15B	108.0
C7—N1—C1	104.76 (12)	C15—C16—H16A	109.5
C7—N2—C6	106.56 (12)	C15—C16—H16B	109.5
C7—N2—C17	126.45 (12)	H16A—C16—H16B	109.5
C6—N2—C17	125.80 (13)	C15—C16—H16C	109.5
C23—N3—C20	114.78 (18)	H16A—C16—H16C	109.5
C23—N3—C19	123.57 (18)	H16B—C16—H16C	109.5
C20—N3—C19	121.59 (17)	N2—C17—C18	110.95 (13)
N1—C1—C2	129.84 (14)	N2—C17—H17A	109.4
N1—C1—C6	109.94 (12)	C18—C17—H17A	109.4
C2—C1—C6	120.21 (13)	N2—C17—H17B	109.4
C3—C2—C1	117.26 (15)	C18—C17—H17B	109.4
C3—C2—H2A	121.4	H17A—C17—H17B	108.0
C1—C2—H2A	121.4	C17—C18—C19	112.78 (15)
C2—C3—C4	121.49 (14)	C17—C18—H18A	109.0
C2—C3—C14	121.05 (15)	C19—C18—H18A	109.0
C4—C3—C14	117.46 (14)	C17—C18—H18B	109.0
C5—C4—C3	121.84 (14)	C19—C18—H18B	109.0
C5—C4—H4A	119.1	H18A—C18—H18B	107.8
C3—C4—H4A	119.1	N3—C19—C18	112.58 (14)
C4—C5—C6	116.48 (15)	N3—C19—H19A	109.1
C4—C5—H5A	121.8	C18—C19—H19A	109.1
C6—C5—H5A	121.8	N3—C19—H19B	109.1
N2—C6—C1	105.75 (12)	C18—C19—H19B	109.1
N2—C6—C5	131.54 (14)	H19A—C19—H19B	107.8
C1—C6—C5	122.71 (13)	N3—C20—C21	103.2 (2)
N1—C7—N2	112.96 (12)	N3—C20—H20A	111.1
N1—C7—C8	125.86 (13)	C21—C20—H20A	111.1
N2—C7—C8	121.17 (13)	N3—C20—H20B	111.1
C13—C8—C9	119.76 (13)	C21—C20—H20B	111.1
C13—C8—C7	120.35 (13)	H20A—C20—H20B	109.1
C9—C8—C7	119.84 (13)	C22—C21—C20	105.7 (2)
C10—C9—C8	120.56 (13)	C22—C21—H21A	110.6
C10—C9—H9A	119.7	C20—C21—H21A	110.6
C8—C9—H9A	119.7	C22—C21—H21B	110.6
O5—C10—C9	125.15 (13)	C20—C21—H21B	110.6
O5—C10—C11	115.23 (12)	H21A—C21—H21B	108.7
C9—C10—C11	119.62 (13)	C21—C22—C23	106.0 (2)
O4—C11—C12	123.67 (13)	C21—C22—H22A	110.5
O4—C11—C10	117.02 (13)	C23—C22—H22A	110.5
C12—C11—C10	119.31 (13)	C21—C22—H22B	110.5
C11—C12—C13	120.81 (13)	C23—C22—H22B	110.5
C11—C12—H12A	119.6	H22A—C22—H22B	108.7
C13—C12—H12A	119.6	O3—C23—N3	125.0 (2)
C8—C13—C12	119.90 (14)	O3—C23—C22	126.9 (2)
C8—C13—H13A	120.1	N3—C23—C22	108.1 (2)
C12—C13—H13A	120.1	O5—C24—H24A	109.5
O2—C14—O1	122.90 (16)	O5—C24—H24B	109.5
O2—C14—C3	123.72 (18)	H24A—C24—H24B	109.5
O1—C14—C3	113.38 (14)	O5—C24—H24C	109.5
C16—C15—O1	111.0 (2)	H24A—C24—H24C	109.5
C16—C15—H15A	109.4	H24B—C24—H24C	109.5
O1—C15—H15A	109.4	H2W1—O1W—H1W1	105 (2)
			
C7—N1—C1—C2	−179.26 (15)	C8—C9—C10—C11	0.9 (2)
C7—N1—C1—C6	0.55 (16)	O5—C10—C11—O4	−1.7 (2)
N1—C1—C2—C3	−179.84 (15)	C9—C10—C11—O4	177.88 (14)
C6—C1—C2—C3	0.4 (2)	O5—C10—C11—C12	178.61 (14)
C1—C2—C3—C4	0.5 (2)	C9—C10—C11—C12	−1.8 (2)
C1—C2—C3—C14	−179.84 (13)	O4—C11—C12—C13	−178.67 (15)
C2—C3—C4—C5	−0.9 (2)	C10—C11—C12—C13	1.0 (2)
C14—C3—C4—C5	179.39 (14)	C9—C8—C13—C12	−1.7 (2)
C3—C4—C5—C6	0.4 (2)	C7—C8—C13—C12	175.77 (14)
C7—N2—C6—C1	1.43 (15)	C11—C12—C13—C8	0.7 (2)
C17—N2—C6—C1	169.57 (13)	C15—O1—C14—O2	3.1 (3)
C7—N2—C6—C5	−179.21 (16)	C15—O1—C14—C3	−177.2 (2)
C17—N2—C6—C5	−11.1 (3)	C2—C3—C14—O2	−173.74 (17)
N1—C1—C6—N2	−1.25 (16)	C4—C3—C14—O2	5.9 (2)
C2—C1—C6—N2	178.58 (13)	C2—C3—C14—O1	6.5 (2)
N1—C1—C6—C5	179.31 (14)	C4—C3—C14—O1	−173.82 (15)
C2—C1—C6—C5	−0.9 (2)	C14—O1—C15—C16	161.8 (3)
C4—C5—C6—N2	−178.83 (15)	C7—N2—C17—C18	77.86 (19)
C4—C5—C6—C1	0.4 (2)	C6—N2—C17—C18	−87.97 (18)
C1—N1—C7—N2	0.40 (17)	N2—C17—C18—C19	−173.95 (13)
C1—N1—C7—C8	−178.76 (14)	C23—N3—C19—C18	−108.7 (2)
C6—N2—C7—N1	−1.19 (17)	C20—N3—C19—C18	68.2 (2)
C17—N2—C7—N1	−169.23 (14)	C17—C18—C19—N3	63.3 (2)
C6—N2—C7—C8	178.01 (13)	C23—N3—C20—C21	−7.6 (3)
C17—N2—C7—C8	10.0 (2)	C19—N3—C20—C21	175.20 (18)
N1—C7—C8—C13	67.7 (2)	N3—C20—C21—C22	13.8 (3)
N2—C7—C8—C13	−111.44 (17)	C20—C21—C22—C23	−15.0 (3)
N1—C7—C8—C9	−114.92 (18)	C20—N3—C23—O3	178.6 (3)
N2—C7—C8—C9	65.99 (19)	C19—N3—C23—O3	−4.3 (3)
C13—C8—C9—C10	0.8 (2)	C20—N3—C23—C22	−1.7 (3)
C7—C8—C9—C10	−176.60 (14)	C19—N3—C23—C22	175.38 (18)
C24—O5—C10—C9	3.3 (3)	C21—C22—C23—O3	−169.5 (3)
C24—O5—C10—C11	−177.18 (17)	C21—C22—C23—N3	10.8 (3)
C8—C9—C10—O5	−179.58 (15)		

### Hydrogen-bond geometry (Å, °)

**Table:**

*Cg*4 is the centroid of the C8–C13 benzene ring.

**Table:**

*D*—H···*A*	*D*—H	H···*A*	*D*···*A*	*D*—H···*A*
O1W—H2W1···O2^i^	0.85 (3)	2.06 (3)	2.879 (2)	164 (2)
O1W—H1W1···N1^ii^	0.95 (3)	1.90 (3)	2.8416 (19)	176 (2)
O4—H1O4···O1W	0.88 (3)	1.80 (3)	2.675 (2)	169 (3)
C16—H16A···O5^iii^	0.96	2.44	3.380 (3)	166.
C20—H20B···Cg4	0.97	2.86	3.750 (3)	153

Symmetry codes: (i) −*x*, −*y*+1, −*z*+1; (ii) −*x*+1, −*y*+1, −*z*+1; (iii) *x*−1, *y*, *z*−1.
